# Dynamic Response and Fatigue Study of BFRP-Reinforced Concrete Slabs Under Random Wave Loading

**DOI:** 10.3390/ma19050880

**Published:** 2026-02-26

**Authors:** Jinlin Huang, Leyuan Jin, Jianwei Zhang, Kelei Cao, Zixu Hu

**Affiliations:** 1 Guangdong Research Institute of Water Resources and Hydropower, Guangzhou 510610, China; hjlcivil@163.com; 2 School of Water Conservancy, North China University of Water Resources and Electric Power, Zhengzhou 450046, China; 15937374497@163.com (L.J.); caokelei456@163.com (K.C.); 18814507709@163.com (Z.H.)

**Keywords:** basalt-fiber-reinforced concrete, fatigue performance, random wave loading, dynamic characteristics

## Abstract

To investigate the dynamic response patterns of basalt-fiber-reinforced concrete slabs under random wave loads, this study characterized wave characteristics based on the random wave theory. Numerical simulations of wave loads were conducted using the Morrison equation, and an analytical model for basalt-fiber-reinforced concrete slabs was established. The research systematically examined the influence mechanisms of two key factors—effective wave period and incident angle—on the dynamic properties of such components. The results indicate that when the effective wave period increases from 7 s to 11 s, the peak displacement, peak stress, peak strain, and stress in the basalt-fiber reinforcement of the slab decrease by 12.79 mm, 0.93 MPa, 130 με, and 229.25 MPa, respectively. The growth rate of the component’s dynamic response first increases and then decreases as the effective wave period shortens. When the wave incidence angle increased from 18° to 90°, the peak displacement, peak stress, peak strain, and stress in the basalt-fiber reinforcement of the concrete slab increased by 17.87 mm, 1.32 MPa, 155 με, and 297.97 MPa, respectively. The growth rate of the component’s dynamic response exhibited a continuous increase with the increasing wave incidence angle. At an incidence angle of 18°, the values of the aforementioned four indicators were 576%, 213%, 52.5%, and 46% higher than those under the 90° condition, respectively. The findings of this study provide theoretical support and data references for elucidating the dynamic response patterns of basalt-fiber-reinforced concrete-slab structures under varying wave-loading conditions and for conducting fatigue performance research.

## 1. Introduction

Breakwaters, bridges, offshore platforms, and other marine engineering structures operate long-term in complex marine environments, continuously subjected to dynamic loads such as waves, which can easily induce structural fatigue damage. If such damage is not effectively prevented and controlled, it may lead to complete structural failure, potentially causing significant economic losses and casualties. Wave loads induce periodic vibrations in structures. Under prolonged cyclic action, localized fatigue damage accumulates, ultimately leading to structural failure. Therefore, accurately assessing fatigue damage induced by wave dynamics during the design phase of marine structures is crucial for ensuring engineering safety. Basalt-fiber-reinforced polymers (BFRPs), characterized by superior mechanical properties and corrosion resistance, have seen expanding applications in marine engineering in recent years [1,2,3]. Against this backdrop, in-depth investigations into the durability and fatigue resistance of basalt-fiber-reinforced concrete structures hold significant theoretical value and practical engineering implications for advancing material upgrades and safety design in marine engineering structures.

The primary methods currently used to calculate wave forces include the characteristic wave method, probability distribution method, spectral analysis method, and wave simulation method. Liang Zuodong et al. [4] employed the P-M (Pierson–Moscowitz) spectrum and Bretschneider spectrum to simulate random waves. Erling Calvert Dolve et al. [5] simulated wave dynamic loads using volume of fluid (VOF) technology, examining the effects of random waves under varying wave heights and frequencies. Park J.C. and Uno Y [6] established a three-dimensional viscous random wave numerical flume to reproduce fully nonlinear multi-directional wave trains, discussing the hydrodynamics experienced by a forward-moving vessel under such wave conditions. Changxin Tang et al. [7] employed numerical simulation techniques to develop a numerical wave-flow model for extreme wave–superstructure interactions on box-girder bridges, comparing predictions with experimental data. Bo Huang et al. [8] conducted a series of fluid–structure interaction experiments and numerical simulations to more accurately investigate the wave forces and dynamic response of the superstructure of a rotatable box girder under extreme wave conditions. Sangmin L. et al. [9] utilized theoretical solutions such as the Morison equation to predict wave forces on offshore structures with vertical cylindrical shapes. Zheng Zhongqiang et al. [10] employed the Morison equation to calculate wave forces on offshore platform structures. Hong Fei Mao et al. [11] obtained hydrodynamic coefficients by solving the Morison equation and provided fitting curves. Ze Shi et al. [12] established structural finite elements using ANSYS, performed a circumferential integration of dynamic water pressure on the wet surface of caisson foundations, and derived wave loads distributed along water depth. Thus, wave simulation methods represent a relatively accurate and widely accepted approach in studying the effects of irregular wave loads on marine structures. This study will employ the Morison equation, using an improved P-M spectrum as the target simulation spectrum, and apply the linear superposition method to simulate wave loads on basalt-fiber-reinforced concrete slabs.

Fatigue damage analyses of engineering structures have garnered widespread attention since frequent component failures observed following the Industrial Revolution. German engineer Wöhler laid the foundational groundwork in this field, with his S-N curve and fatigue limit providing crucial guidance for subsequent developments. Khan Rizwan A et al. [13] investigated the design of welded structures under fatigue limit conditions, employing linear or bilinear S-N curves to predict their service life. Zhang Long et al. [14] studied the fatigue failure of circumferential welds in SCRs under harsh environments and complex platform motion conditions, providing accurate fatigue damage predictions for welded joints by applying the structural stress fatigue theory and S-N curve methods. Wu Bo et al. [15] proposed a novel damage prediction method based on broadband stochastic processes. Combining a standard spectral design with different PSD shapes and Monte Carlo simulations, they derived empirical formulas for expected damage rates. Jingyi Ding et al. [16] investigated the fatigue resistance of floating wind turbines under complex marine environmental loads, performing fatigue damage assessments using the direct probability integration method. Compared with traditional Monte Carlo simulations, this approach demonstrated improved accuracies and computational efficiencies. Hui Long et al. [17] established a finite element model for a beam structure containing breathing cracks, employing the Walker equation for fatigue crack damage assessment to predict the fatigue life of steel. Haiyang Ge et al. [18,19] constructed a standard stress spectrum to determine distribution parameters, weighting factors, and distribution correction coefficients related to the spectral width parameter, enabling real-time fatigue-loss prediction for marine structures in the frequency domain. They also proposed a dual-modal response model for two well-separated Gaussian random processes, establishing a Rayleigh distribution-based model for fatigue analyses of marine structures. M. Lurohman Mamin et al. [20] evaluated wave-loading effects by discretizing theoretical wave spectra. Long Zhang et al. [21] derived the stresses and power spectral densities of modal coordinate responses by analyzing modal structural stresses, thereby establishing effective fatigue stress parameters for analysis. This demonstrates the indispensable role of fatigue damage analyses in the operational performance of engineering structures.

Current research on the fatigue behavior of BFRP-reinforced concrete-slab structures under random wave loads remains insufficient. Studies on the dynamic loading of fiber reinforcement under conventional wave loads typically employ either the characteristic wave method or conventional spectral analysis. However, the characteristic wave method oversimplifies wave randomness, while conventional spectral analysis struggles to accurately describe the coupling effects between BFRP-reinforced concrete slabs and random waves. Concurrently, fatigue assessment methods based on steel cannot be directly applied to BFRP-reinforced concrete slabs, which exhibit pronounced brittle characteristics. To address these challenges, this study proposes a novel approach integrating the random wave theory, the Morison equation, and refined finite element modeling. This enables the synergistic analysis of BFRP-reinforced concrete slabs under corrosive environments and dynamic loading.

A high-precision three-dimensional finite element model of BFRP-reinforced concrete slabs was constructed, enabling real-time coupling between BFRP reinforcement corrosion behavior and wave loading. By simulating various wave types—including wave height, period, incident angle, and water depth—the study investigated their effects on the stress, deformation, and damage characteristics of BFRP-reinforced concrete slabs. The study examines how the dynamic response of these materials evolves under varying mix proportions and environmental conditions, particularly focusing on the effects on stress, deformation, and damage characteristics. Based on simulation results, the dynamic effects and damage evolution patterns of BFRP-reinforced concrete slabs under different wave types and performance conditions are determined. This enables an assessment of the performance of BFRP-reinforced concrete slabs under varying wave conditions and environmental influences. In summary, this research aims to deepen the understanding of wave loading effects on BFRP-reinforced concrete slab performance to enhance the design and safety of marine and coastal engineering structures. Through simulation analysis, it provides more accurate data for practical applications.

## 2. Experimental Study on BFRP Reinforcement

To clarify the design parameters and performance characteristics of basalt-fiber composites as reinforcement components, this study experimentally tested and evaluated their mechanical properties. This provides a parameter foundation for subsequent simulation research and optimizes their practical application in specific engineering projects.

This study utilized the basalt-fiber-composite reinforcement of model BFCB-8-A-ER, manufactured and supplied by Sichuan Aerospace Wuyuan Composite Materials Co., Ltd., Chengdu, China. Experimental results indicate that this material exhibits an average measured tensile strength of 970 MPa, an average tensile modulus of elasticity of 49.0 GPa, an average elongation at break of 2.8%, an average ultimate bending angle of 49.3°, an average measured alkali-resistance retention rate of 95.8%. and an average acid-resistance retention rate of 92.6%.

### Experimental Study on the Mechanical Properties of BFRP in Marine Environments

**Figure 1:**
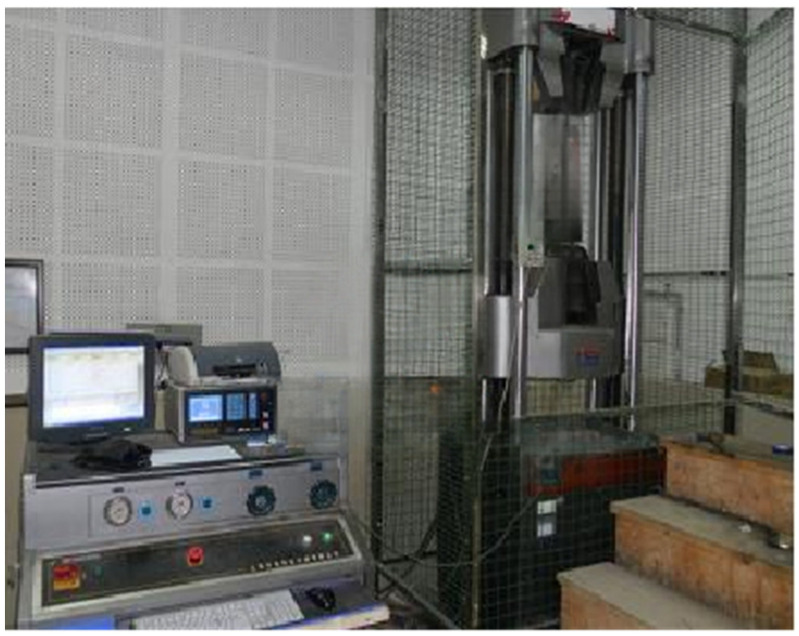
CNC testing machine in the experiment.

**Figure 2:**
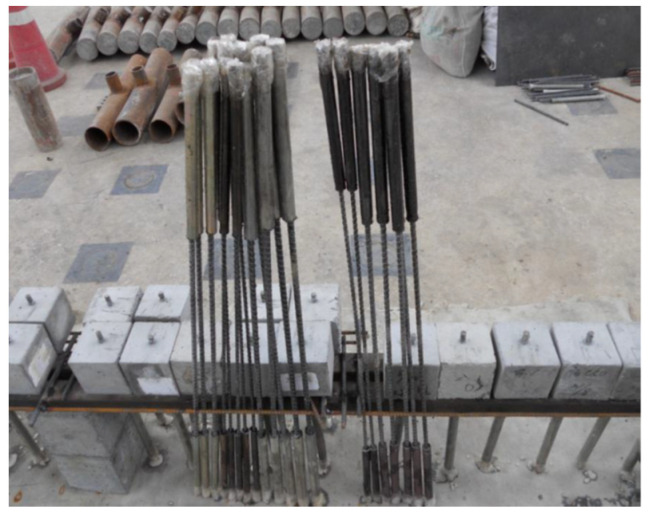
Anchored specimens.

For fiber materials, tensile testing is a critical experiment for evaluating their mechanical properties, with the results closely related to stress analysis. Tensile tests on basalt-fiber composite tendons were conducted using a CNC hydraulic universal testing machine (as shown in Figure 1), with digital extensometers precisely recording elongation. Seamless steel tubes served as anchor heads during testing. Epoxy resin mixed with quartz sand was used as a curing agent to anchor the basalt-fiber-composite tendons (as shown in Figure 2).

Specimens, prepared and cured for over one week, were placed between the testing machine’s grips. A digital extensometer was mounted at the specimen’s midpoint. Load was applied at a rate of 2 mm per minute until specimen failure occurred. The maximum tensile stress at failure and failure characteristics were recorded. Unlike steel, basalt-fiber-composite reinforcement exhibits no distinct yield stage during tensile loading. The specimen’s force–displacement curve increases linearly to the maximum force before sudden failure. To ensure testing equipment safety, immediately remove the extensometer when specimen stress reaches 600 MPa. Utilize the equipment’s embedded displacement acquisition system to precisely record strain values before specimen failure occurs.

**Figure 3:**
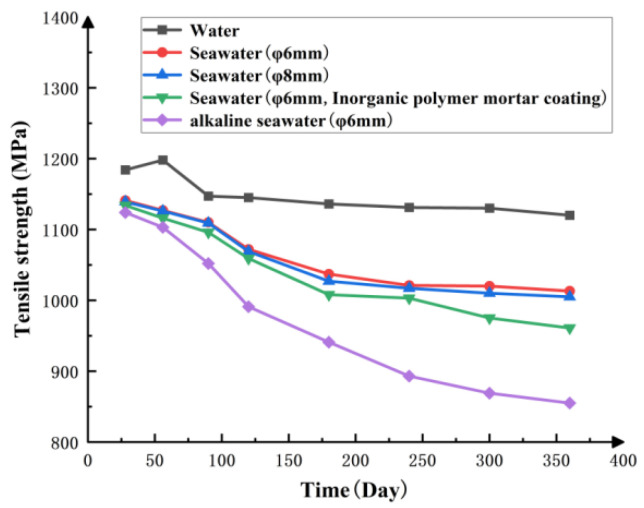
Changes in tensile strength of BFRP bars in different corrosive media.

To investigate the tensile strength variation characteristics of BFRP tendons under various corrosive environments, BFRP tendon samples with a diameter of Φ6 mm were selected and subjected to prolonged immersion in tap water, simulated seawater environments (including direct immersion and immersion after encapsulation with inorganic polymer mortar), and alkaline seawater. The experiment aimed to systematically reveal the evolution of BFRP bars’ mechanical properties under different corrosive media. Specific results are shown in Figure 3. As illustrated, after tap water immersion, the tensile strength of BFRP bars exhibited an initial increase followed by a decrease, eventually stabilizing at a constant value. In contrast, immersion in alkaline seawater caused a significant and pronounced decline in tensile strength. Immersion in simulated seawater similarly caused a marked decline in tensile strength. However, encapsulation with inorganic polymer mortar effectively mitigated the subsequent tensile strength degradation of BFRP bars during service. During the corrosion process, the tensile strength of BFRP bars exhibits a dynamic pattern: an initial sharp decline followed by gradual attenuation. This phenomenon indicates that when the corrosive medium penetrates to a specific depth within the BFRP bar, an equilibrium state is reached. At this stage, chemical reaction products accumulate inside the BFRP bar, effectively hindering further acceleration of the erosion process and causing the erosion rate to level off. Comparing 6 mm and 8 mm diameter BFRP bars encased in inorganic polymer mortar, the 8 mm diameter bars exhibit more pronounced declines in tensile strength and ultimately achieve lower tensile strengths than the 6 mm diameter bars. The tensile performance results obtained from the immersion tests on BFRP bars will be applied to the finite element model simulation analysis in this paper.

## 3. Wave Theory and Wave Simulation and Verification

### 3.1. Random Wave Theory

Current research on wave analysis largely relies on numerical modeling. Engineering approaches treat waves as the superposition of an infinite number of harmonic waves based on the power spectral density of waves. By numerically simulating wave profiles under specific environmental conditions, this process assumes waves as one of the stationary random processes whose characteristics can be described by the superposition of multiple cosine waves with different periods and random initial phases [22]. Therefore, the wave profile function can be expressed as

(1) $$\eta (t)=\sum_{i=1}^{N}a_{i}\cos({\hat{\omega }}_{i}t+\varepsilon_{i})$$

(2) $$\Delta \omega_{i}=\omega_{i-1}-\omega_{i}$$

In the formula, η(t) is wave height–time history, m; Sη(a_2_) is wave target spectrum; ϵ_i_ is initial phase of the i-th constituent wave; Q_1_ is representative frequency, rad, taking a random value between ω_i−1_ ∼ ω_i_; and Q_i_ is the average value between ω_i−1_ ∼ ω_i_, rad.

In different marine environments, the wave loads experienced by offshore structures exhibit significant variations. This characteristic underscores the importance of precisely selecting the appropriate wave theory during analysis and calculation. Currently, wave theories exhibit multidimensional and complex characteristics. However, the most widely applied theories in engineering practices primarily include the solitary wave theory [23] and random wave theory [24]. These theories are highly favored due to their broad applicability and effectiveness in practical engineering scenarios. The influencing factors of waves differ under varying water depths.

Wave theory is primarily categorized into linear waves and nonlinear waves. Within the scope of engineering applications, the selection of an appropriate wave theory must be determined based on specific circumstances. This process emphasizes the flexibility of theoretical selection, aiming to solve practical problems with greater precision. Real-world wave characteristics exhibit high uncertainty and randomness, with parameters such as direction, amplitude, and period displaying irregular variations. Traditional deterministic wave theories, which overlook this randomness and complexity, fail to accurately reflect actual wave behavior. Therefore, to more precisely simulate and understand the dynamic properties of real waves, the more advanced methodology of the stochastic wave theory must be adopted. Grounded in probability statistics, the stochastic wave theory treats sea surface fluctuations as random processes. This approach effectively captures and describes the random variations in waves, thereby providing more precise and practical means for analyzing and predicting wave characteristics. The modified P-M spectrum is selected as the target for wave analysis, namely

(3) $$S_{\eta }\left(\omega \right)=\frac{A}{\omega^{5}}\exp(-B\omega^{-4})$$

(4) $$A=173H_{s}^{2}T_{0.1}^{-4}$$

(5) $$B=691T_{0.1}^{-4}$$

In the formula, S_η_(ω) is wave spectrum, m^2^⋅s, and T_0.1_ is the average period for spectrum calculation, s.

In marine engineering structures, the selection of wave-load calculation methods is typically determined by whether the structure is large-scale or small-scale. For small-scale structures, wave drag and inertia forces are the primary components, whereas for large-scale structures, wave inertia and diffraction forces are the most significant components. In engineering design, wave force calculations employ different methodologies based on structural scale, as the nature of the forces varies significantly with size.

In the design and analysis of marine engineering structures, the selection of methods for calculating wave loads typically depends on the structure’s large-scale characteristics.

This approach considers the overall behavior of the structure and the impact of wave forces on the entire structure. In contrast, for small-scale structures or when more detailed analyses of specific regional stress conditions are required, distinguishing between these two approaches facilitates selecting the most appropriate computational strategy for different design requirements. For small-scale structures, wave drag and inertia forces play the primary role. For large-scale structures, wave inertia and diffraction forces become the decisive factors.

The subject of this study is the small-scale structure of BFRP-reinforced concrete slabs. According to the Morison equation, the concrete slab itself has no significant effect on wave motion. The wave action on the slab is divided into drag forces generated by viscous effects and inertial forces generated by added mass effects. Wave force f_H_ at height z of the plate is

(6) $$f_{H}=f_{D}+f_{t}$$

(7) $$f_{D}=C_{D}\rho \frac{U^{2}\left(t\right)}{2}=\frac{1}{2}C_{D}\rho A\left|U(t)\right|U(t)$$

(8) $$f_{t}=2\pi \rho a^{2}\frac{\mathrm{dU}}{\mathrm{dt}}=C_{M}\rho V\frac{\mathrm{dU}}{\mathrm{dt}}$$

In the formula, A is the projected area of the unit plate height perpendicular to the direction of wave propagation, m^2^; U is the horizontal velocity of a wave-like water particle at any height z along the plate’s axial position, m/s; dU/dt is the horizontal acceleration of a wave-like water particle at any height z along the plate’s axial position, m/s^2^; V is the volume per unit length of the plate body, V = πα^2^; a is the radius of the cylinder, m; ρ is the density of seawater, kg/m^3^; C_D_ is the drag coefficient; and C_M_ is the inertia coefficient. Using the Morison equation and wave surface Equation (1) as transfer functions, the wave force spectrum expression can be derived. The wave force spectrum at height z is given by

(9) $$S_{F}\left(\omega \right)={\left(\frac{1}{2}\frac{\gamma }{g}C_{D}D\sqrt{\frac{8}{\pi }}\sigma_{U}\omega \frac{\cos h\;kz}{\sin h\;kd}\right)}^{2}S_{\eta }\left(\omega \right)+{\left(\frac{\gamma }{g}C_{M}A\omega^{2}\frac{\cos h\;kz}{\sin h\;kd}\right)}^{2}S_{\eta }\left(\omega \right)$$

### 3.2. Theories Related to Fatigue Analysis

**Figure 4:**
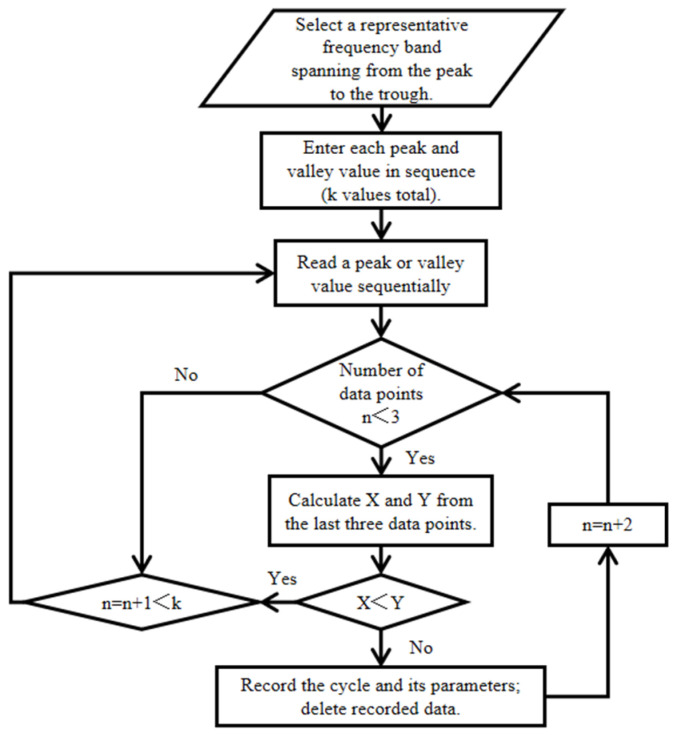
Rain-flow counting procedure diagram.

The rain-flow counting method is a widely used technique in engineering, indispensable for fatigue life assessment. By rotating the strain–time history curve by 90°, with the time axis oriented vertically downward and data values progressing along the time axis, the resulting pattern resembles a series of stacked roofs, akin to rain flowing downward. Hence, this method is named the rain-flow counting method [25]. This method simulates material memory properties by counting time-series data during loading, aligning with real mechanical principles, and thus gaining widespread acceptance. It is also applicable to the conditions described in this paper. Based on the rain-flow counting rules, a program was developed, with the flowchart shown in Figure 4. Here, X and Y represent the calculation ranges. For the last three data points, the absolute value of the difference between the first and second points is Y and the absolute value of the difference between the second and third points is X.

The S-N curve depicts the relationship between the number of cycles required for material failure and the stress amplitude applied. Consequently, a structure’s fatigue resistance is closely tied to this curve. S-N curves are typically determined through cumulative fatigue testing in laboratories, established by progressively increasing the number of loading cycles. When selecting S-N curve parameters for fatigue assessment, multiple factors must be comprehensively considered, including, but not limited to, structural design and material properties. Referencing prior experimental and theoretical calculations, and considering that the dynamic response stress amplitude in this study’s concrete is less than 0.7 times the ultimate strength of the concrete used, the S-N curve adopted to calculate the compressive fatigue strength of concrete in this study is as follows [26].

(10) $$S_{\max}=1.0505-0.0656\mathrm{lg}N_{f}$$

In components subjected to cyclic loading, assuming stresses are independent of each other, fatigue damage values are calculated using the linear superposition method. Once accumulated damage reaches a specific threshold, the component is deemed to have failed due to fatigue. The linear fatigue cumulative damage theory distinguishes between the number of cycles under different stress amplitudes Δσ_1_, Δσ_2_,Δσ_3_ …, denoted as N_1_, N_2_, N_3_, …, and the actual number of cycles experienced, denoted as n_1_, n_2_, n_3_… Defining the damage component as D_i_, the specific value of this damage component is the ratio of the number of cycles corresponding to each stress amplitude to the number of cycles required to reach fatigue failure. Assuming that stress variations generated within the structure under different loads are regarded as mutually independent events, the local damage caused by each cyclic load can similarly be viewed as independent events that do not influence one another. Thus, the total damage equals the sum of the damage from each individual cyclic load. That is,

(11) $$D=D_{1}+D_{2}+\ldots +D_{i}=\frac{n_{1}}{N_{1}}+\frac{n_{2}}{N_{2}}+\ldots +\frac{n_{i}}{N_{i}}=\sum_{i}\frac{n_{i}}{N_{i}}$$

In the formula, n_i_ denotes the actual number of cycles under the stress amplitude at the i-th level; N_i_ denotes the allowable number of cycles to fatigue failure at the stress amplitude of the i-th level, calculated from the S-N curve; and i denotes the total number of stress amplitude values corresponding to all operating conditions involved in damage calculation.

### 3.3. Wave Loading Verification

**Figure 5:**
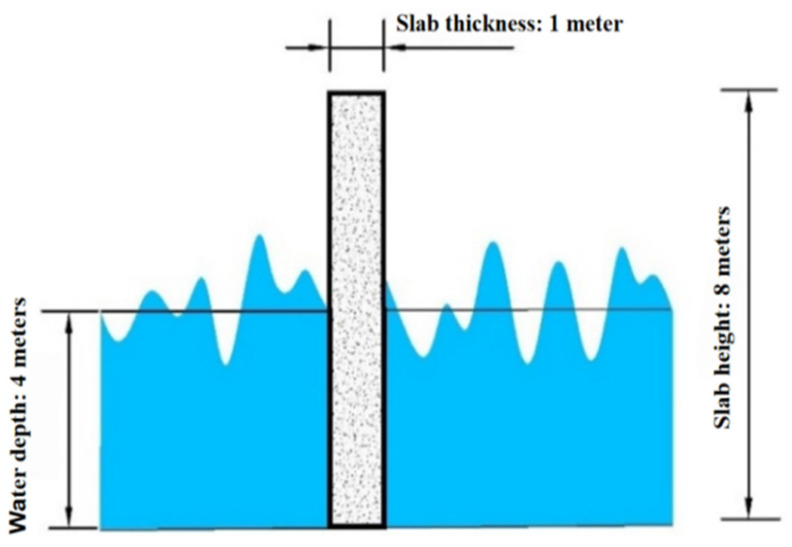
Random wave load loading diagram.

The concrete slab is positioned in water at a depth of 4 m, with a design effective wave height of 2 m. The wave action direction is perpendicular (90°) to the slab. The random wave load diagram is shown in Figure 5. The approximate relationship between wave height and period is shown in Table 1.

**Figure 6:**
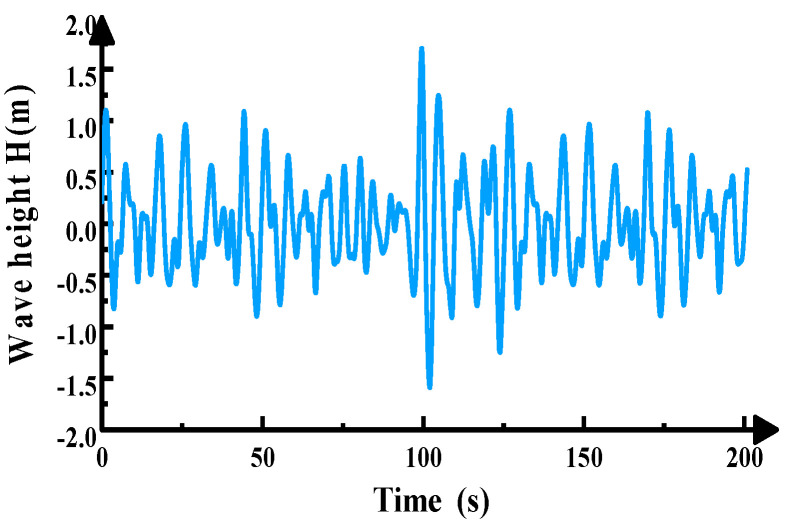
Height–time history diagram of random wave load.

**Figure 7:**
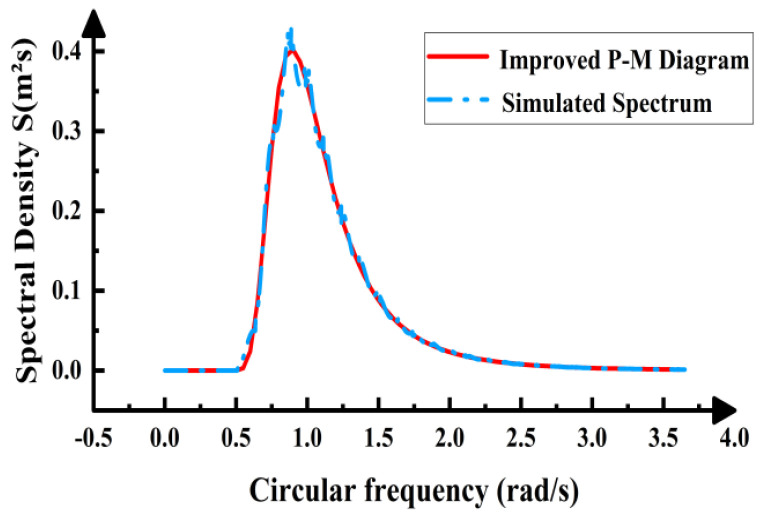
Modified P-M spectra.

The effective wave height H_s_ corresponds to a wave period T_s_ = 6.1 s, yielding a corresponding wavelength L = T × (gd)^1/2^ = 42.7 m. Since b/L = 0.14 < 0.2, the concrete slab is classified as a small-scale structure, and the Morrison equation is employed to simulate the wave loading. Based on Equation (1) and the modified P-M spectrum, the wave height–time history curve was obtained using the harmonic superposition method. This revealed that the wave height exhibited non-periodic variations over time with no discernible pattern. This primarily stems from the wave height–time history curve being generated from random phases, imparting inherent randomness to the curve. The autocorrelation function of this curve undergoes the Fourier transform to yield the simulated spectrum. Comparison with the target spectrum is shown in Figure 6 and Figure 7. The figures reveal excellent agreement between the simulated and target spectrum curves, with a maximum error of only 6%. This validates the accuracy of the curve results, confirming the suitability of this spectrum for wave load simulation.

### 3.4. Establishment of Simulation Model

**Figure 8:**
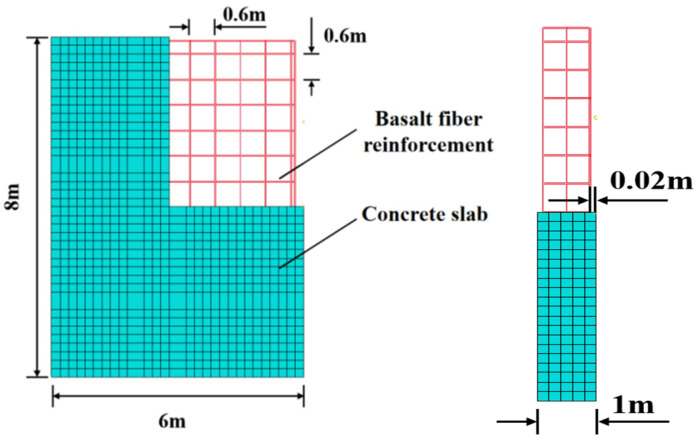
Detail structure of BFRP-reinforced concrete slab.

To investigate the dynamic response of basalt-fiber-reinforced polymer (BFRP) concrete slabs under random wave loads, a three-dimensional finite element model of a BFRP-reinforced concrete slab was established (as shown in Figure 8). This model applies only fixed constraints at the bottom of the slab; no constraints are applied to the sides. The concrete-slab model dimensions were 6 m wide × 8 m high × 1 m thick. BFRP reinforcement was modeled using linear elements, while the concrete slab employed three-dimensional solid elements. The mesh primarily consisted of regular hexahedral elements, totaling 13,645 nodes and 12,536 elements across the entire model. The interaction between the concrete slab and BFRP reinforcement was established through embedded constraints. The concrete model utilized a concrete damage–plasticity (CDP) constitutive model, incorporating the following parameters for plastic behavior: expansion angle (Ψ), eccentricity (ϵ), biaxial-to-uniaxial stress ratio (fb0/fc0), shape factor (K), and viscosity (μ), with values of 33°, 0.1, 1.165, 0.66667, and 0.2, respectively. The BFRP reinforcement model employed an elastic–plastic constitutive relationship. The tensile yield strength was selected based on the experimental value simulated after 28 days of immersion in seawater. Other physical and mechanical parameters are detailed in Table 2. All results originate from experimental studies conducted under the research project on the application technology of fiber-reinforced-composite concrete in water conservancy projects, which was led by the Guangdong Provincial Water Resources and Hydropower Research Institute.

### 3.5. Scheme Design and Path Selection

To qualitatively investigate the influence patterns of random wave loads on BFRP-reinforced concrete slabs, the following operational conditions were established based on design data and historical hydrological conditions from a southern coastal region. Conditions I–V were formulated using different effective periods as variables, while conditions VI–X were developed using varying incident angles as variables. The ten loading conditions were grouped into two sets to separately examine the dynamic response of BFRP-reinforced concrete slabs under varying wave parameters, as shown in Table 3.

**Figure 9:**
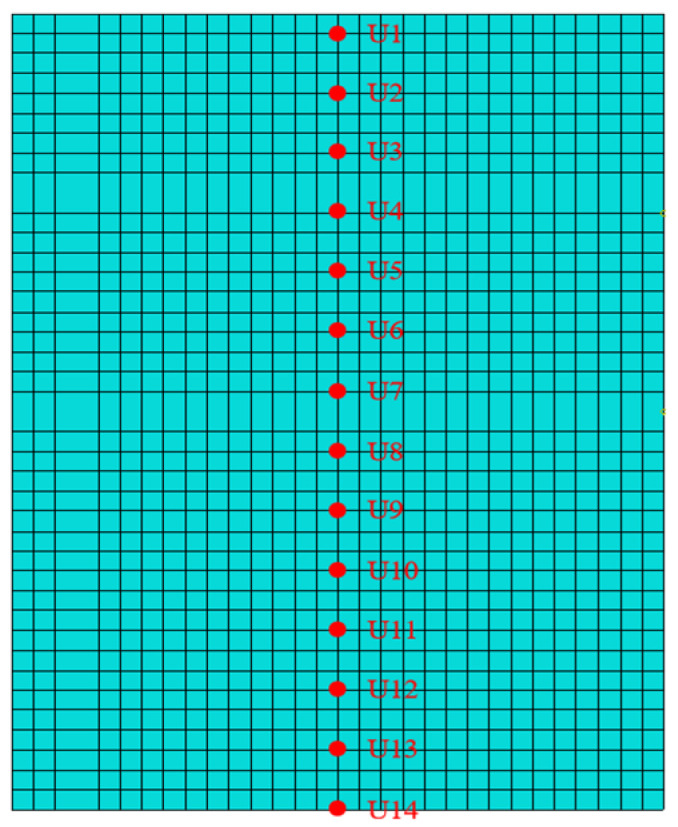
Concrete slab path 1.

**Figure 10:**
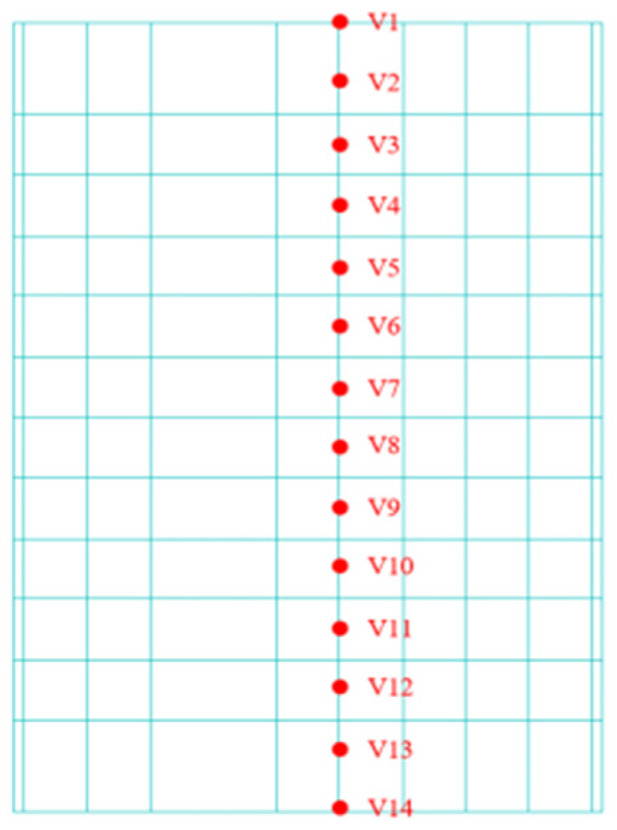
Concrete slab path 2.

**Figure 11:**
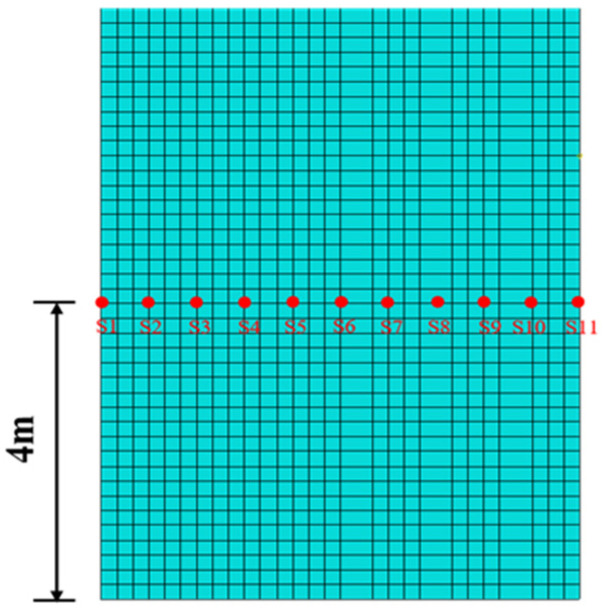
Concrete slab path 3.

**Figure 12:**
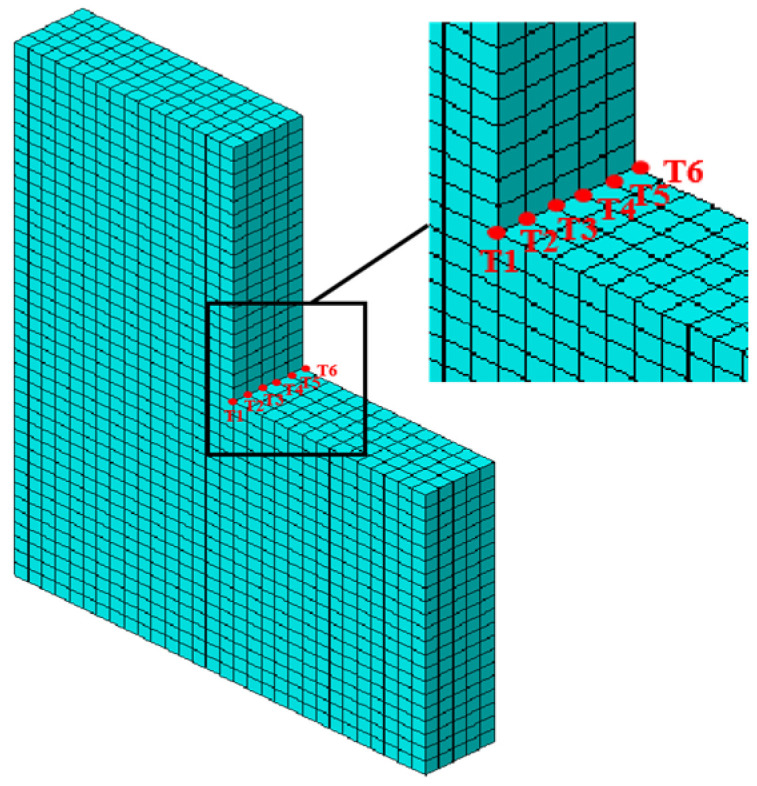
Concrete slab path 4.

To investigate the dynamic response of BFRP-reinforced concrete slabs under various vertical loading conditions with different wave parameters, paths 1 and 2 were selected along the slab surface and perpendicular to the BFRP reinforcement, respectively, as shown in Figure 9 and Figure 10. To investigate the mechanical properties of BFRP-reinforced concrete slabs under various wave conditions in the transverse and thickness directions, a transverse path 3 was selected at the 4 m water level of the slab. Simultaneously, a path 4 extending in the thickness direction was derived from point S6 on the transverse path to examine the slab’s mechanical characteristics along the thickness direction, as shown in Figure 11 and Figure 12.

## 4. Effects of Variations in Different Wave Parameters on the Mechanical Properties of BFRP-Reinforced Concrete Slabs

### 4.1. Effect of Effective Cycle Variation on the Mechanical Properties of BFRP Concrete Slabs

#### 4.1.1. Effect of Effective Period Variation on Concrete Slab Displacement

**Figure 13:**
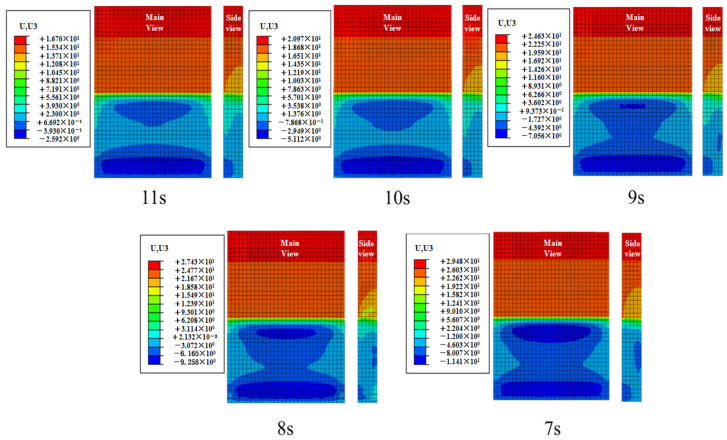
Displacement contour of concrete slab under different effective periods.

**Figure 14:**
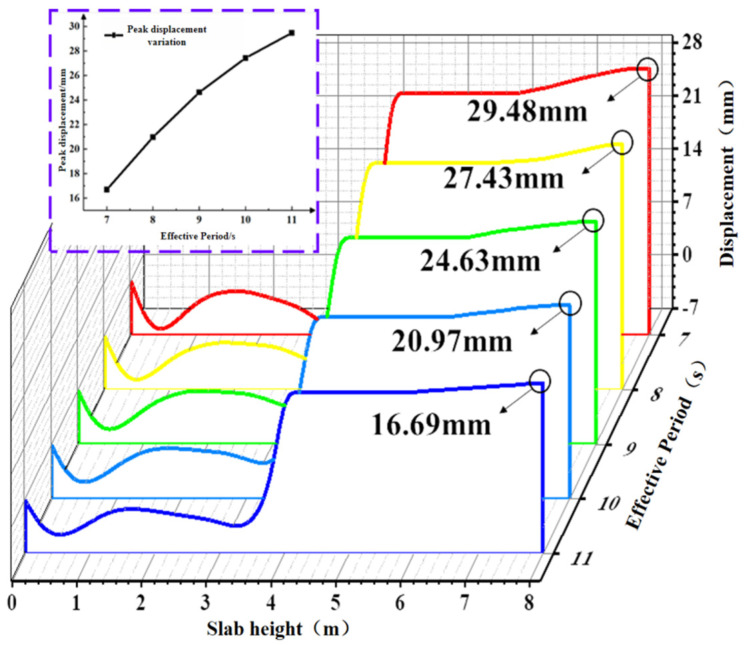
Displacement values of plates under different effective periods.

The displacement contour plot of the concrete slab, plotted based on the extreme case at the point of maximum displacement during the total simulation duration of 1200 s, is shown in Figure 13. Based on the aforementioned extreme condition of the concrete slab, path 1 was selected to extract the peak displacement in the Z-direction from the contour plot, as depicted in Figure 14. The figure indicates that the peak displacement of the slab increases as the effective period shortens, with the rate of increase gradually slowing down. As the period shortens from 11 s to 7 s, the peak displacements of the concrete slab are 16.69 mm, 20.97 mm, 24.63 mm, 27.43 mm, and 29.48 mm, respectively. Under the 7 s condition, the peak displacement of the concrete slab increases by 76.6% compared with the 11 s condition.

#### 4.1.2. Effect of Effective Period Variation on Concrete Slab Stress

**Figure 15:**
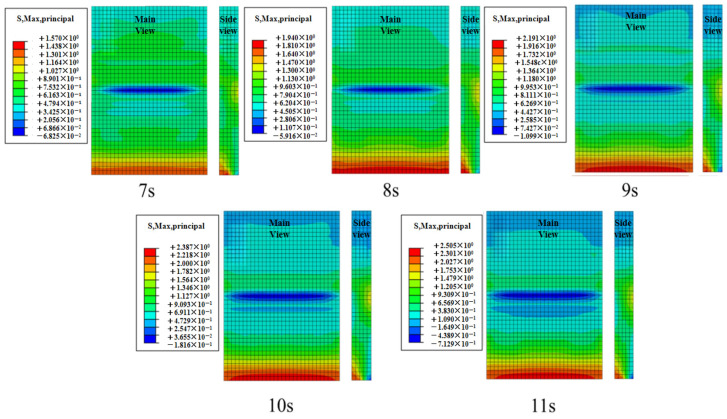
Stress contour diagram of concrete slab under different effective periods.

**Figure 16:**
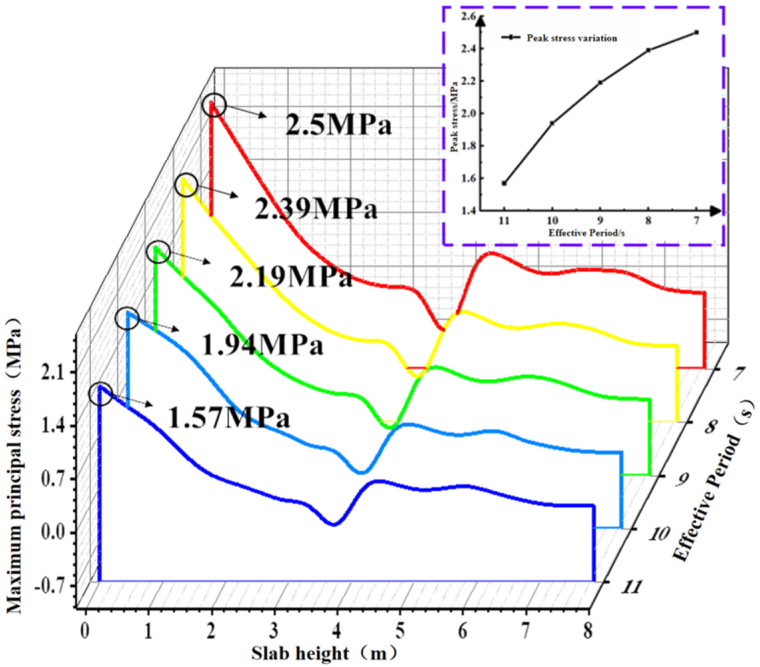
Maximum principal stress values of the plate under different effective periods.

Figure 15 shows the contour plots of the maximum principal stress in the concrete slab at different effective wave heights during the total simulation duration of 1200 s. Path 1 was selected to extract the maximum principal stress of the slab from the contour plots, as shown in Figure 16. The peak stress increases as the effective period shortens, with its rate of increase gradually slowing down. As the period shortened from 11 s to 7 s, the peak values of the maximum principal stress in the concrete were 1.57 MPa, 1.94 MPa, 2.19 MPa, 2.39 MPa, and 2.50 MPa, respectively. Under the 7 s condition, the peak stress in the concrete slab increased by 59.2% compared with the 11 s condition.

#### 4.1.3. Effect of Effective Period Variation on Concrete Slab Strain

**Figure 17:**
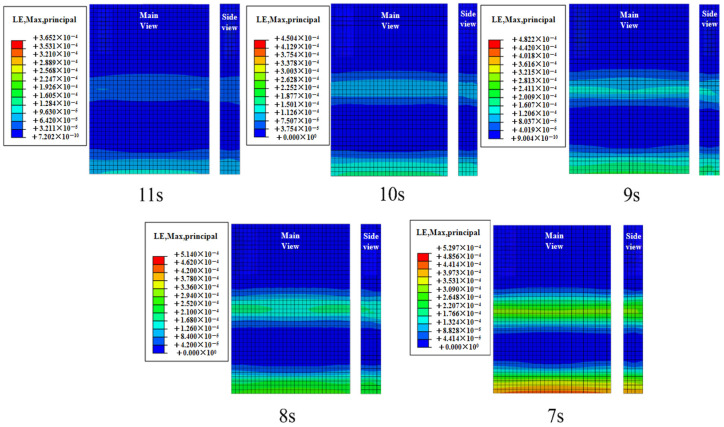
Strain contour diagram of concrete slab under limit conditions under different effective periods.

**Figure 18:**
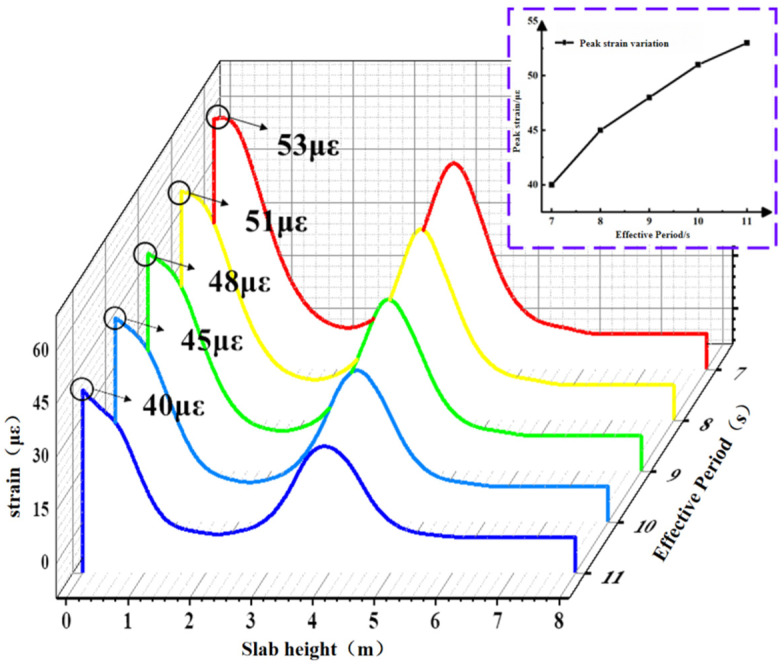
Strain diagram of the plate under different effective cycles.

The contour plot of the maximum concrete slab strain during the 1200 s simulation period is shown in Figure 17. Path 1 was selected to extract the slab strain from the contour plot, as depicted in Figure 18. Peak strain exhibits a gradual increase trend as the effective period shortens. As the period shortens from 11 s to 7 s, the peak concrete slab strains are 365 με, 450 με, 480 με, 510 με, and 530 με, respectively. The peak strain under the 7 s effective period condition increases by 32.5% compared with the 11 s condition.

#### 4.1.4. Effect of Effective Cycle Variation on BFRP Reinforcement Stress

**Figure 19:**
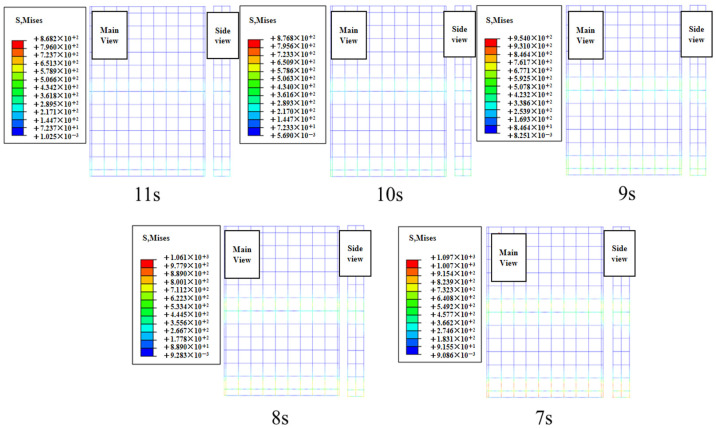
BFRP tendon contour diagram of the limit case under different effective periods.

**Figure 20:**
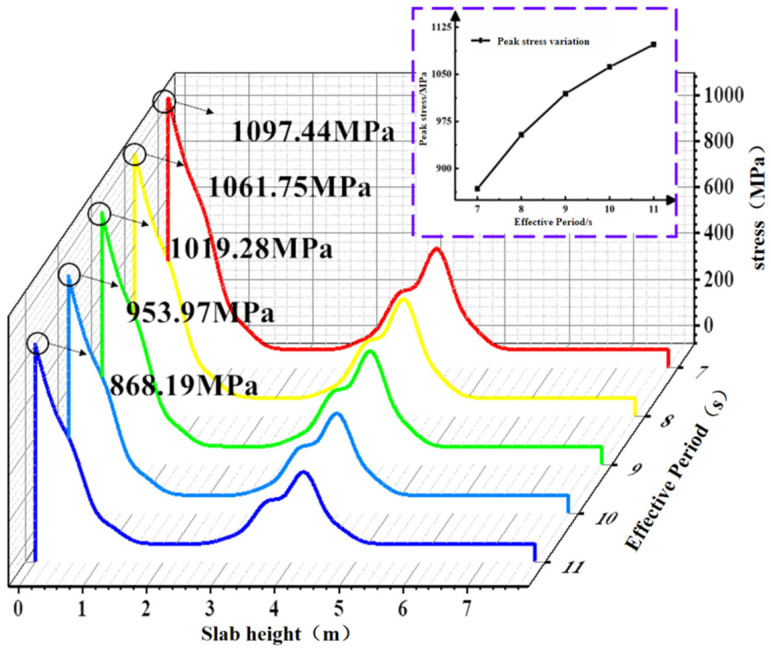
BFRP tendon stress diagram under different effective periods.

To investigate the variation in BFRP reinforcement stress values under different effective wave heights, a stress contour map of the BFRP reinforcement stress under extreme conditions was plotted at the time point with the maximum stress in the concrete slab during the total simulation duration of 1200 s, as shown in Figure 19. The stress values under extreme conditions for the BFRP reinforcement on path 1 were extracted. As shown in Figure 20, the stress in the BFRP reinforcement exhibits a gradually increasing trend as the effective period shortens. As the period decreases from 11 s to 7 s, the peak stresses in the BFRP reinforcement are 868.19 MPa, 953.97 MPa, 1019.28 MPa, 1061.75 MPa, and 1097.44 MPa, respectively. Under the 7 s condition, the peak stress of BFRP reinforcement increased by 26.4% compared with the 11 s condition.

**Figure 21:**
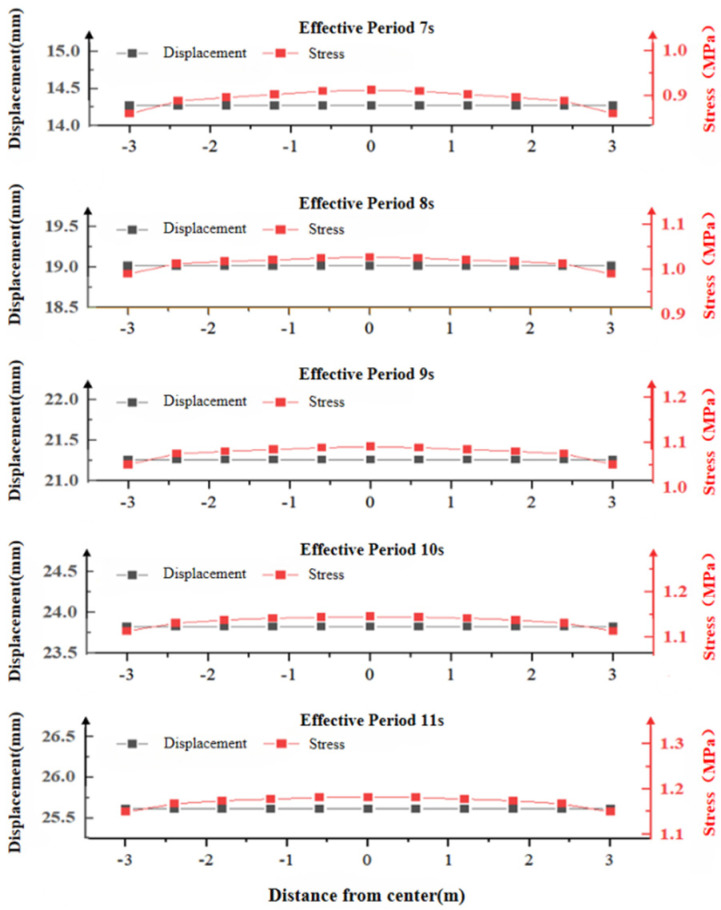
Relationship between the maximum principal stress and displacement of the concrete slab in path 4.

**Figure 22:**
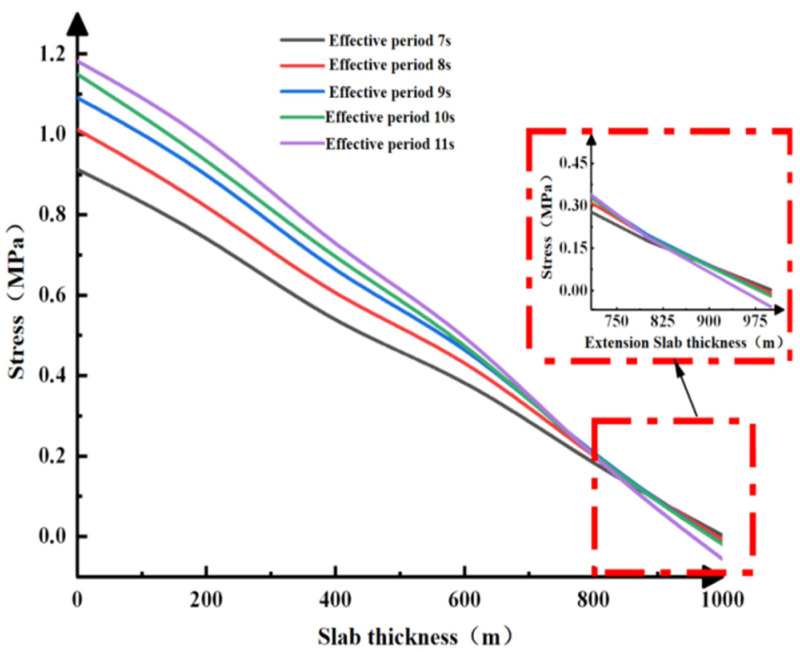
Relationship between the maximum principal stress and displacement of concrete in path 3.

Stress curves and plate displacement curves in the Z-direction under different cycles along path 3, along with the maximum principal stress values of the concrete slab under path 4, are shown in Figure 21 and Figure 22. Similarly, the lateral displacement values did not change with variations in the maximum principal stress. At path point 4, the maximum principal stress at the 0 m contact surface between the concrete slab and the wave increases with the effective cycle, though the rate of increase gradually slows. As the distance from the wave contact surface increases, the maximum principal stress decreases sharply. Therefore, subsequent incidence angle conditions are not discussed further.

### 4.2. Effect of Incidence Angle Variation on the Mechanical Properties of BFRP Concrete Slabs

#### 4.2.1. Effect of Incident Angle Variation on Concrete Slab Displacement

**Figure 23:**
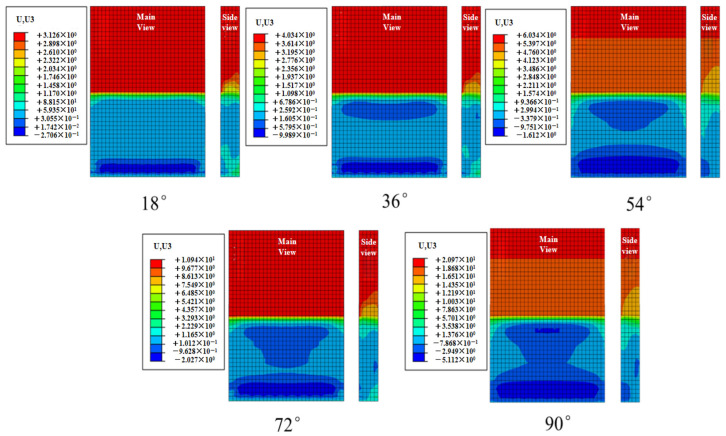
Displacement contour of concrete slab under limit cases at different angles of incidence.

**Figure 24:**
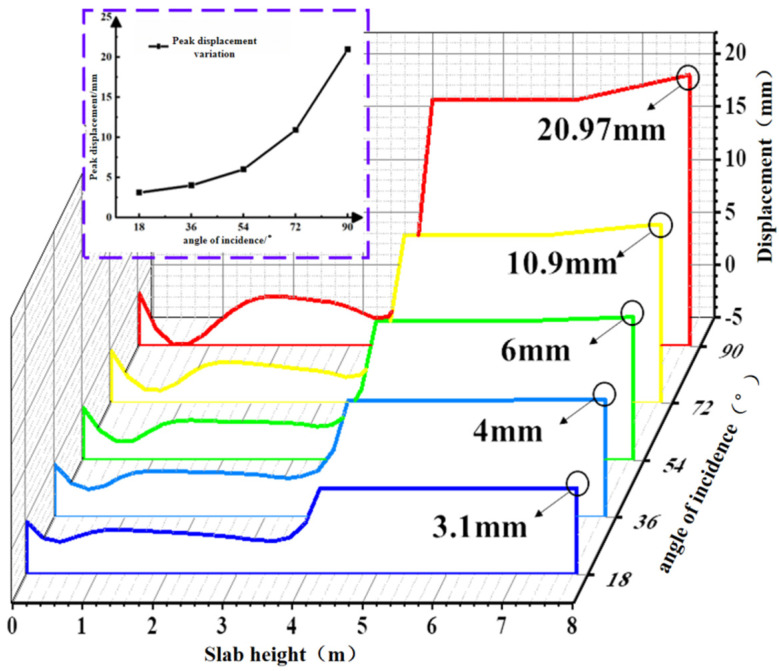
Displacement diagram of the lower plate at different angles of incidence.

Due to the random nature of wave loads, the displacement contour plot of the concrete slab is generated by selecting the maximum displacement point during the entire simulation duration of 1200 s, as shown in Figure 23. Based on the aforementioned extreme condition of the concrete slab, path 1 was selected to extract the peak Z-direction displacement from the displacement contour map, as shown in Figure 24. The figure indicates that the peak displacement of the slab increases with the incident angle, exhibiting a gradually increasing slope. A steep rise occurs between 72° and 90°. The peak displacements at different incidence angles are 3.1 mm, 4 mm, 6 mm, 10.9 mm, and 20.97 mm, respectively. Under the 90° condition, the peak displacement of the concrete slab increases by 576.4% compared with the 18° condition.

#### 4.2.2. Effect of Incidence Angle Variation on Concrete Slab Stress

**Figure 25:**
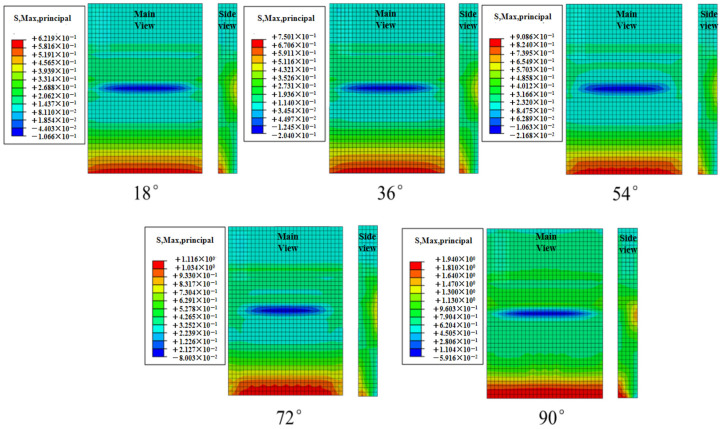
Stress contours of concrete slabs under limit cases at different angles of incidence.

**Figure 26:**
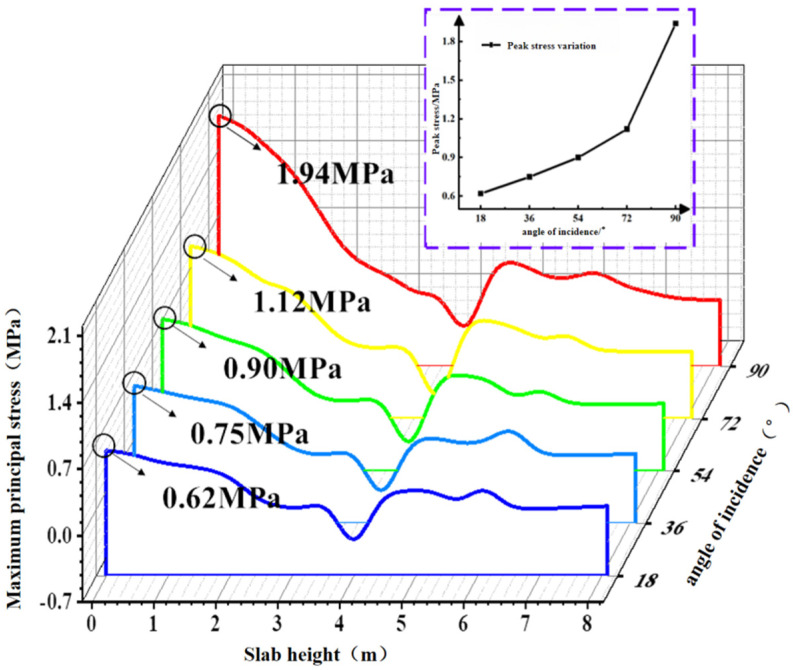
Maximum principal stress values of the plate at different angles of incidence.

Figure 25 shows the contour plots of the maximum principal stress in the concrete slab at different effective wave heights during the total simulation duration of 1200 s. Path 1 was selected to extract the maximum principal stress values from the contour plots, as depicted in Figure 26. The peak stress increases with the incident angle, and the slope of this variation gradually increases. The peak values of the maximum principal stress at different incidence angles are 0.62 MPa, 0.75 MPa, 0.90 MPa, 1.12 MPa, and 1.94 MPa, respectively. Under the 90° condition, the peak stress of the concrete slab increases by 212.9% compared with the 18° condition.

#### 4.2.3. Effect of Incident Angle Variation on Concrete Slab Strain

**Figure 27:**
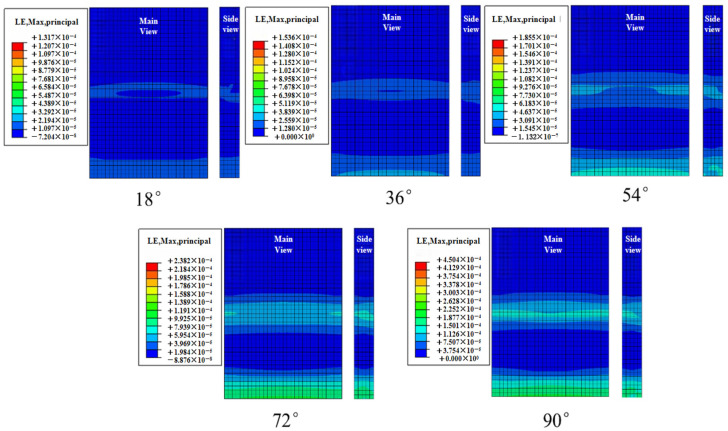
Strain contour diagram of concrete slab under limit conditions under different effective periods.

**Figure 28:**
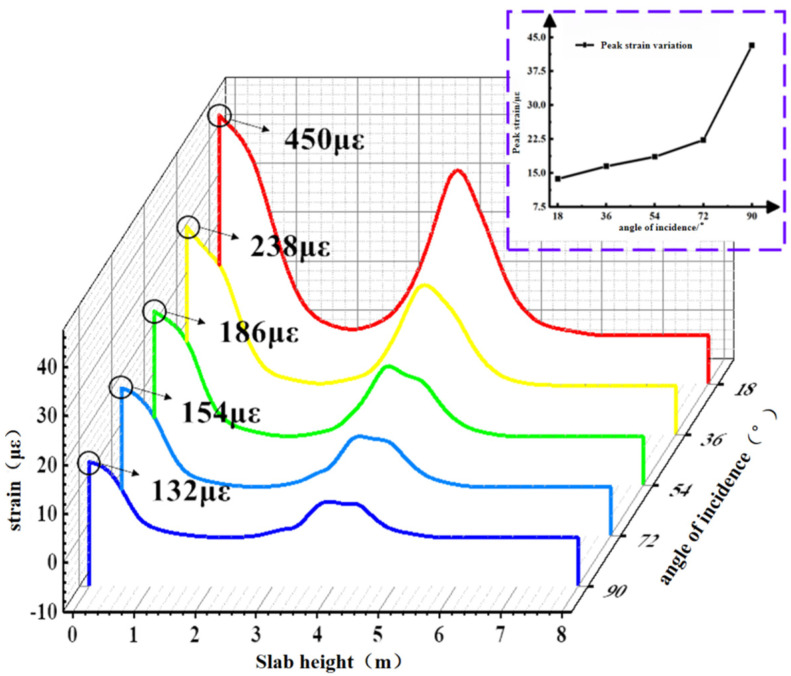
Strain diagram of the lower plate at different angles of incidence.

The contour plot of the maximum strain on the concrete slab during the 1200 s simulation is shown in Figure 27. Path 1 was selected to extract the slab strain from the contour plot, as depicted in Figure 28. The slope of the peak strain variation in the concrete slab gradually increases with the incident angle. The peak strains at different incident angles are 132 με, 154 με, 186 με, 238 με, and 450 με, respectively. Under the 90° condition, the peak strain of the concrete slab increases by 240.1% compared with the 18° condition.

#### 4.2.4. Effect of Incidence Angle Variation on BFRP Reinforcement Stress

**Figure 29:**
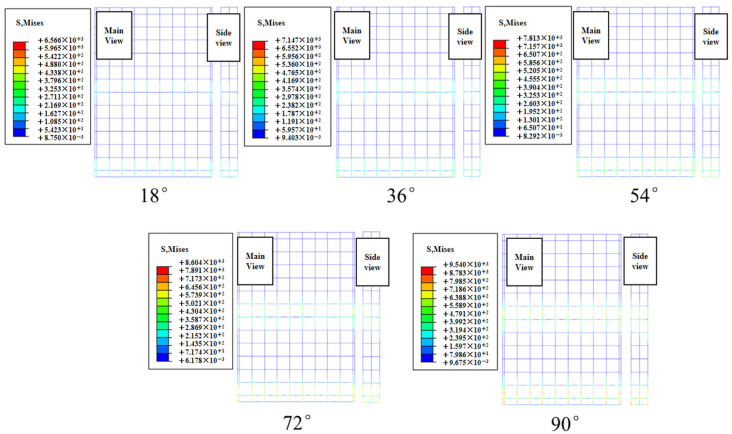
BFRP tendon contour diagram of the limit case under different effective periods.

**Figure 30:**
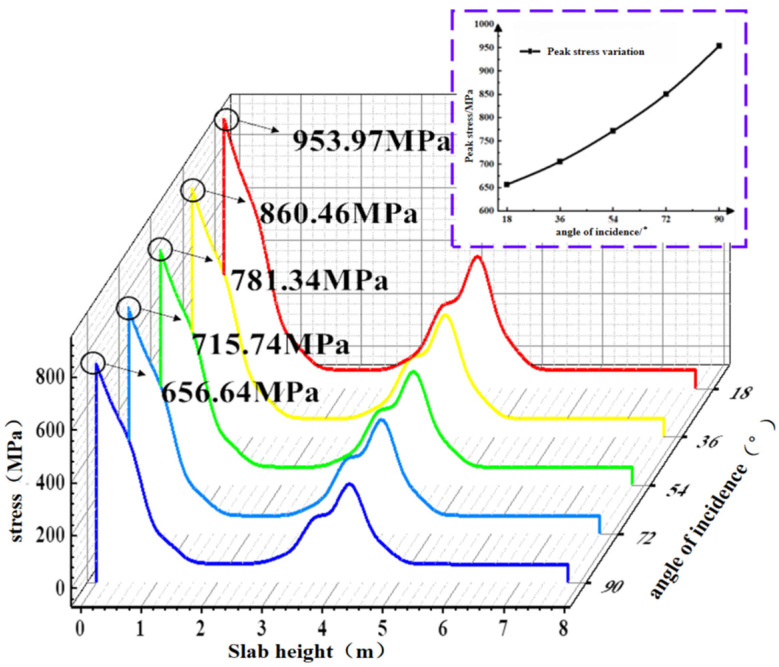
BFRP tendon stress diagram under different effective periods.

To investigate the variation in BFRP reinforcement stress values under different significant wave heights, a stress contour plot of the maximum stress point in the BFRP reinforcement of the concrete slab during the total simulation duration of 1200 s is shown in Figure 29. Extract the peak stress value of the BFRP reinforcement at path point 1. As shown in Figure 30, the stress of the BFRP reinforcement increases with the incident angle, and the rate of change gradually rises. The peak stresses of BFRP tendons under effective wave heights of 1.0 m to 3.0 m are 656.64 MPa, 714.74 MPa, 781.34 MPa, 860.46 MPa, and 953.97 MPa, respectively. Under the 3.0 m condition, the peak stress of BFRP tendons increases by 40.2% compared with the 1.0 m condition.

## 5. Study on Fatigue Properties of Basalt-Fiber-Reinforced Concrete Slabs with Different Wave Elements

### 5.1. Study on Fatigue Properties of Basalt-Fiber-Reinforced Concrete Slabs Under Different Effective Life Cycles

#### 5.1.1. Stress–Time History

This chapter investigates the fatigue damage of basalt-fiber-reinforced concrete slabs under random wave loading, using the basalt-fiber-reinforced slabs with varying wave parameters calculated in Section 3 as the research subject. Specific parameters and the modeling process are detailed in Section 2. As previously described, the dynamic response of these concrete slabs under different wave parameters was studied, analyzing the effects of varying effective periods and incident angles, with comparative analyses conducted. Building upon the calculations in Section 3, compressive stress–time histories were extracted from the concrete slab at three locations: the top, middle, and bottom. For these extracted compressive stress-time histories, fatigue damage calculations were performed on them. This chapter conducts fatigue damage calculations for the operating conditions mentioned in Section 3 and provides a comparative analysis of their fatigue performance.

**Figure 31:**
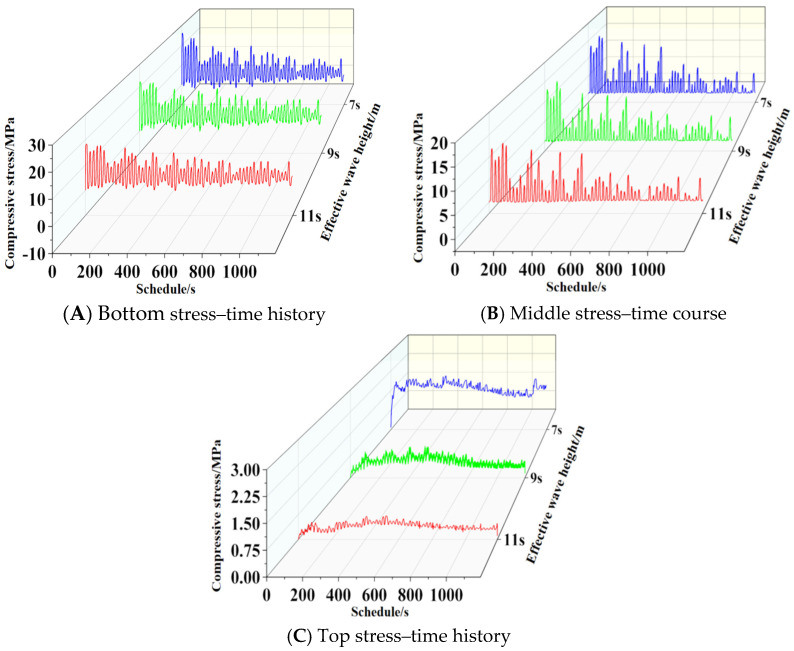
Stress–time history curves for different effective periods.

To investigate the influence of different effective periods on the fatigue behavior of basalt-fiber-reinforced concrete structures, three operating conditions were selected from Section 3.1: effective periods of 7 s, 9 s, and 11 s; an incident angle of 90°; and a water depth of 4 m. Following calculations, compressive stress–time histories were extracted for the slab bottom (1 m), mid-section (4 m), and top (7 m) under different loading conditions. Results are shown in Figure 31.

#### 5.1.2. Fatigue Analysis Results

**Figure 32:**
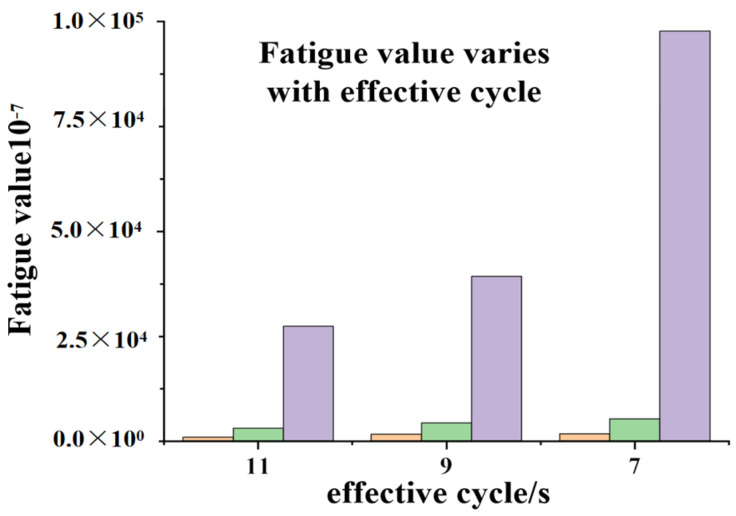
Histograms of fatigue values for different effective cycles.

The stress time history was calculated using the obtained data., the damage conditions at three locations—the center, top, and bottom of the plate—under varying stresses can be calculated. Table 4 presents the fatigue damage results. For easier visualization, Figure 32 illustrates the fatigue damage at the center and top of the plate under different effective wave heights.

The fatigue values for the bottom, middle, and top sections of the concrete slab under a 1200 s stress history were calculated programmatically. As shown in the table, the fatigue values for the bottom, middle, and top sections under different effective cycles are not on the same order of magnitude. This is because the material fatigue curve used is a logarithmic function, and the magnitude of the stress level S_max_ significantly affects the final fatigue value. This also explains why the fatigue value at the bottom of the slab under an effective cycle of 7 s is 102% higher than that under an effective cycle of 11 s. Furthermore, the fatigue value generated at an effective cycle of 7 s is greater than that at 11 s. This occurs because shorter wave cycles result in more frequent impacts on the concrete slab. Therefore, in actual engineering applications, the energy generated by short waves should not be overlooked compared with that of long waves. Furthermore, the stress–time curve in Figure 32 reveals that during the 9 s effective cycle, the compressive stress generated by wave impact on the concrete slab exhibits a sudden increase at a specific initial moment, surpassing the maximum compressive stress observed in the 7 s cycle. However, the 9 s cycle ultimately does not yield a higher fatigue value than the 7 s cycle. This indicates that although isolated instantaneous values are higher during the simulation, these transient peaks do not cause lower-energy waves to induce greater structural fatigue than higher-energy waves. The magnitude of fatigue damage is determined by the overall stress profile: larger overall stress amplitudes result in greater fatigue values for the structure.

The calculation results show that under the 1200 s stress cycle, maximum fatigue occurs at the bottom of the plate under the 7 s effective period condition, with a fatigue value of 2.978 × 10^−7^. Repeated application of the 1200 s stress cycle sequence until reaching the fatigue limit value 1 would require 127 years. This duration represents only the expected fatigue failure lifespan of the concrete slab under wave forces, indicating that wave-induced damage is a critical factor that cannot be overlooked during the service life of a cross-sea bridge. Compared with the actual marine environment, the discussion here pertains solely to fatigue calculations under normal wave conditions. In this model, we have considered only the effects of wave loading. However, incorporating the high corrosion factors common in marine environments would significantly increase the anticipated stress amplitude, leading to more severe fatigue damage. Furthermore, in actual marine settings, cross-sea bridges endure not only wave loads but also multiple environmental stresses such as currents, wind forces, seismic activity, and salt spray corrosion. The combined effects of these factors on marine structures accelerate the fatigue damage process, thereby impacting structural safety and service life. Therefore, marine structure design must fully account for and quantify the impact of these environmental loads. By employing advanced materials, optimizing structural design, and implementing effective maintenance strategies, we can ensure the long-term stable operation of marine structures and enable them to effectively withstand diverse environmental challenges.

### 5.2. Study on Fatigue Properties of Basalt-Fiber-Reinforced Concrete Slabs at Different Incidence Angles

#### 5.2.1. Stress–Time History

**Figure 33:**
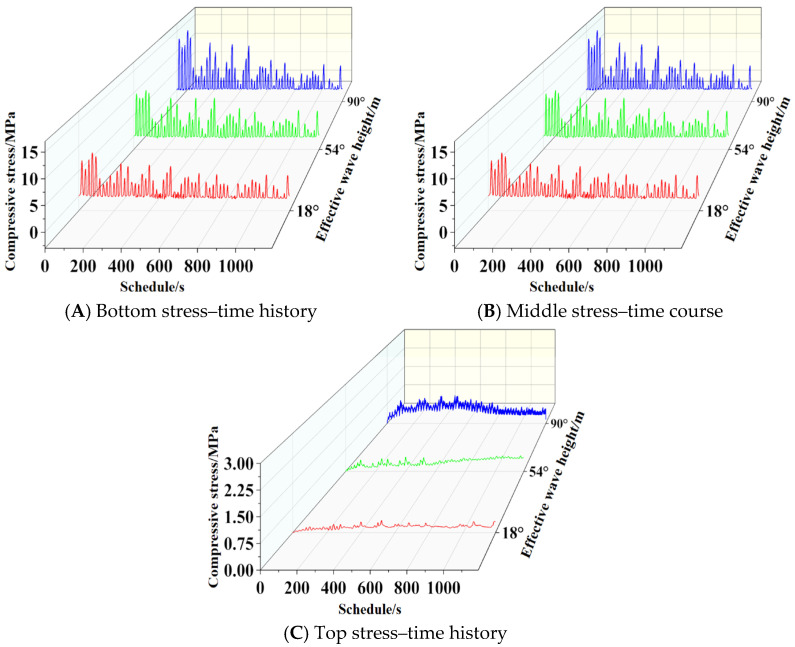
Time history curves of stress at different angles of incidence.

To investigate the influence of different effective periods on the fatigue behavior of basalt-fiber-reinforced concrete structures, three loading conditions were selected from Section 3.2: incident angles of 18°, 54°, and 90°, with an effective wave height of 2 m; an effective period of 10 s; and a water depth of 4 m. Following calculations, the compressive stress–time histories at the bottom (1 m), middle (4 m), and top (7 m) of the slab under different loading conditions were extracted, with results shown in Figure 33.

#### 5.2.2. Fatigue Analysis Results

**Figure 34:**
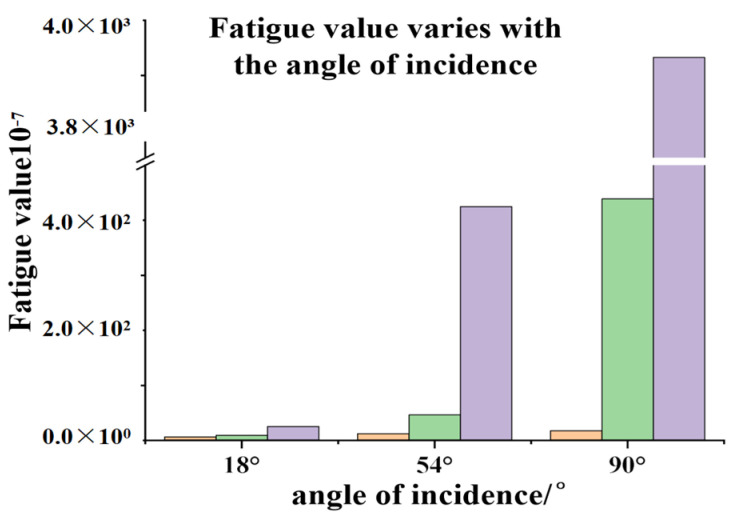
Histograms of fatigue values at different angles of incidence.

By importing the obtained stress history into the program, the damage conditions at three locations, the center and top of the plate bottom—under different stresses—can be calculated. Table 5 presents the fatigue damage calculation results. Figure 34 illustrates the fatigue damage at the center and top of the plate bottom under different effective wave heights.

The fatigue values at the bottom, middle, and top of the concrete slab under a 1200 s stress cycle were calculated through the program. Since the effective wave height for all three different incidence angle conditions is 2 m, even the fatigue values generated at the maximum incidence angle of 90° do not pose a significant threat to the concrete slab. However, as the incidence angle increases, the fatigue values imposed on the slab rise exponentially, far exceeding the fatigue damage inflicted on basalt-fiber-reinforced concrete slabs under different effective cycles. Determining the optimal angle for structural exposure to waves in practical engineering applications warrants further investigation.

## 6. Conclusions

This paper analyzes the mechanical properties and fatigue performance of BFRP-reinforced concrete slabs under different effective periods and incident angles of wave parameters. The research conclusions are as follows:

(1) As the effective wave period shortens, the peak displacement, peak maximum principal stress, peak strain, and BFRP reinforcement stress of the slab all increase non-linearly. The rate of increase first rises and then decreases. When the effective period shortens from 11 s to 7 s, the plate peak displacement changes are 16.69 mm, 20.9 mm, 24.63 mm, 27.43 mm, and 29.48 mm, respectively; the plate peak maximum principal stresses were 1.57 MPa, 1.94 MPa, 2.19 MPa, 2.39 MPa, and 2.50 MPa, respectively; the peak strain variations in the plate were 40 με, 45 με, 48 με, 51 με, and 53 με; and the peak stresses of the BFRP reinforcement were 868.19 MPa, 953.97 MPa, 1019.28 MPa, 1061.75 MPa, and 1097.44 MPa.
(2) As the incident angle increases, the plate’s peak displacement, peak stress, peak strain, and BFRP reinforcement stress all significantly increase, with the rate of change showing a gradually increasing trend. As the incident angle increases from 18° to 90°, the plate peak displacement changes to 3.1 mm, 4 mm, 6 mm, 10.9 mm, and 20.9 mm; the plate peak stress changes to 1.12 MPa, 1.25 MPa, 1.40 MPa, 1.62 MPa, and 1.94 MPa; the plate peak strain changes to 29.5 με, 31.5 με, 34.3 με, 38 με, and 45 με; and the peak stresses in the BFRP reinforcement are 756.64 MPa, 785.74 MPa, 821.34 MPa, 870.46 MPa, and 953.97 MPa, respectively.
(3) Considering the combined effects of the effective period and incidence angle, the incidence angle exerts the most significant influence on the BFRP-reinforced concrete slab, followed by the effective period. It is important to note that shorter effective wave periods exert a more critical influence on the structure. For the horizontal direction of the slab, the magnitude of the maximum principal stress along the transverse path of the concrete slab does not produce displacement differences in the same horizontal plane. Along the thickness direction of the slab, the maximum principal stress value decreases sharply until the maximum principal stress on the wave surface at the back of the slab exhibits no discernible pattern.
(4) Research on the fatigue performance of BFRP-reinforced concrete slabs indicates that instantaneous stress transients do not cause low-energy wave fatigue values to exceed those of high-energy waves. The magnitude of fatigue damage is determined by the overall stress conditions: the greater the overall stress amplitude, the higher the fatigue value inflicted on the structure. As the incident angle increases, the fatigue values induced in the slab rise exponentially, exhibiting a significantly greater rate of increase compared with the fatigue values observed in basalt-fiber-reinforced concrete slabs under different effective periods.

## 7. Prospect

With the increasing exploitation of marine resources, the safety and durability of marine structures have become increasingly critical. Against this backdrop, BFRP reinforcement, as a novel reinforcement material, has demonstrated a broad application potential in marine engineering due to its high strength. This study provides a theoretical basis for the dynamic response, dynamic behavior, and fatigue performance of BFRP-reinforced concrete slabs under wave loading; however, research in this field remains in its developmental stage. Future research can be expanded and deepened in the following directions:

(1) Current studies primarily focus on the impact of wave loads on structures. However, marine structures are typically subjected to the coupled effects of multiple factors, such as wind loads and seismic loads. Future research should incorporate multi-field coupling analysis methods to investigate the combined influence of wave loads and other environmental factors on structural performance under different environmental conditions, thereby enhancing the comprehensiveness and accuracy of structural design.
(2) For calculating fatigue damage in BFRP-reinforced concrete slabs under different operating conditions, it is advisable to select representative waves based on measured wave force data from actual marine environments for simulation. Calculate the corresponding fatigue damage values caused by these measured waves to the structure, ultimately evaluating the cumulative fatigue damage of BFRP-reinforced concrete structures.

In the future, breakwaters can be selected as the subject for applying the random wave theory to compile wave force spectra and wave force time histories. Based on actual project profiles, finite element models of engineering breakwaters can be established for simulation experiments. This approach will demonstrate the applicability of wave action in influencing the dynamic response and fatigue characteristics of BFRP-reinforced concrete and reinforced concrete structures.

## Figures and Tables

**Table 1:** Approximate relationship between wind and waves and cycles.

*H*_1/3_/m	2	3	4	5	6	7	8	9	10
*T*s/s	6.1	7.5	8.7	9.8	10.6	11.4	12.1	12.7	13.2

**Table 2:** Material parameters.

Materials	Density	Modulus of Elasticity	Poisson Ratio	Tensile Strength
C45 concrete	2600 kg/m^3^	32,500 MPa	0.2	/
BFRP(Φ8 mm)	2100 kg/m^3^	500,000 MPa	/	1150 MPa

**Table 3:** Working conditions.

Operating Condition Number	Effective Wave Height/m	Effective Period/s	Incidence Angle/°	Water Depth/m
I	2	11	90	4
II	2	10	90	4
III	2	9	90	4
IV	2	8	90	4
V	2	7	90	4
VI	2	10	18	4
VII	2	10	36	4
VIII	2	10	54	4
IX	2	10	72	4
X	2	10	90	4

**Table 4:** Fatigue values for different.

	Fatigue Damage Value (×10^−7^)		
Wave Conditions	Bottom of the Board	Center of the Board	Top of the Board
11 s	0.0000148	0.0031	1.474
9 s	0.0000177	0.0044	2.183
7 s	0.0000199	0.0053	2.978

**Table 5:** Fatigue values at different angles of incidence.

	Fatigue Damage Value (×10^−7^)		
Wave Conditions	Bottom of the Board	Center of the Board	Top of the Board
18°	0.0000001	0.00009	0.003
54°	0.0000012	0.00046	0.043
90°	0.0000156	0.0044	0.379

## Data Availability

The original contributions presented in this study are included in the article. Further inquiries can be directed to the corresponding author.
